# Supplementary material for: A redox mechanism underlying nucleolar stress sensing by nucleophosmin
Source: Nat Commun. 2016 Nov 25;7:13599. doi: 10.1038/ncomms13599 (PMC5133708; doi:10.1038/ncomms13599)
Supplement: Supplementary Information — Supplementary Figure 1-7, Supplementary Table 1 [file ncomms13599-s1.pdf]

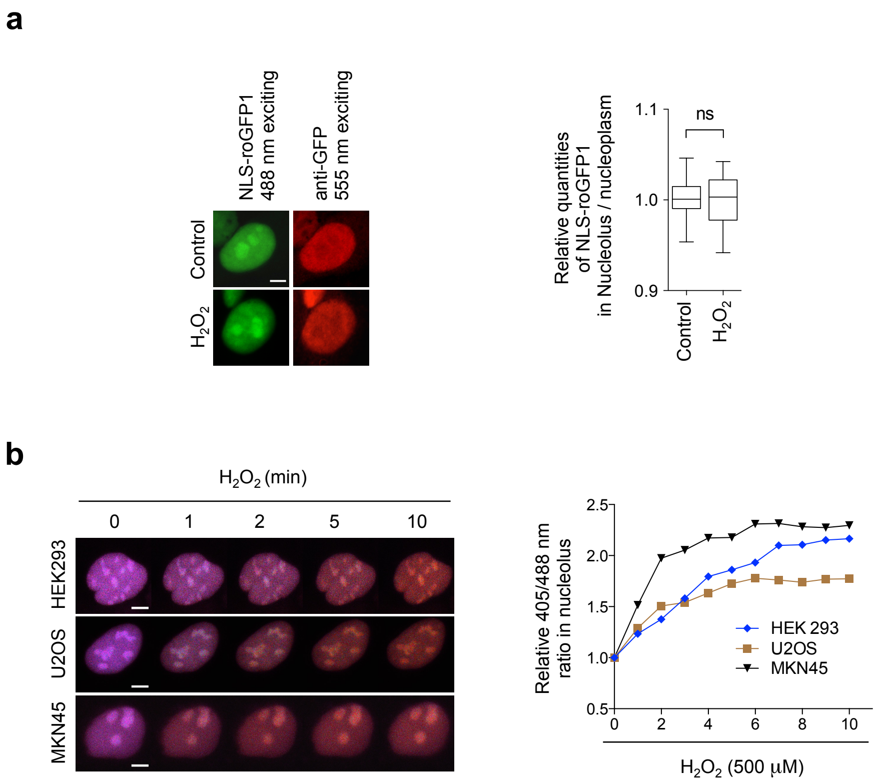

Supplementary Figure 1 | (a) NLS-roGFP1 was expressed in HeLa cells and treated with H_2O_2 (500 μM), and indicated by immunostaining with mouse anti-GFP primary antibody and 555-labelled goat anti-mouse secondary antibody. The 488 nm exciting images indicated the redox states, and the 555 nm exciting images indicated the quantities of NLS-roGFP1 protein. The quantities of NLS-roGFP1 protein in nucleolus *vs.* nucleoplasm are calculated and displayed as box and whisker plots, n=20 cells in each group from three independent experiments, Unpaired t test. **(b)** Nucleolar redox changes of 10 min after H_2O_2 (500 μM) treatment in HEK293, U2OS and MKN45 cell lines. Representative images from two independent experiments and relative 405/488 nm ratio value per minute were displayed. Bars: 5 μm. Data are represented as mean ± s.e.m. ns, no statistical significance. This is related to Fig. 1.

a

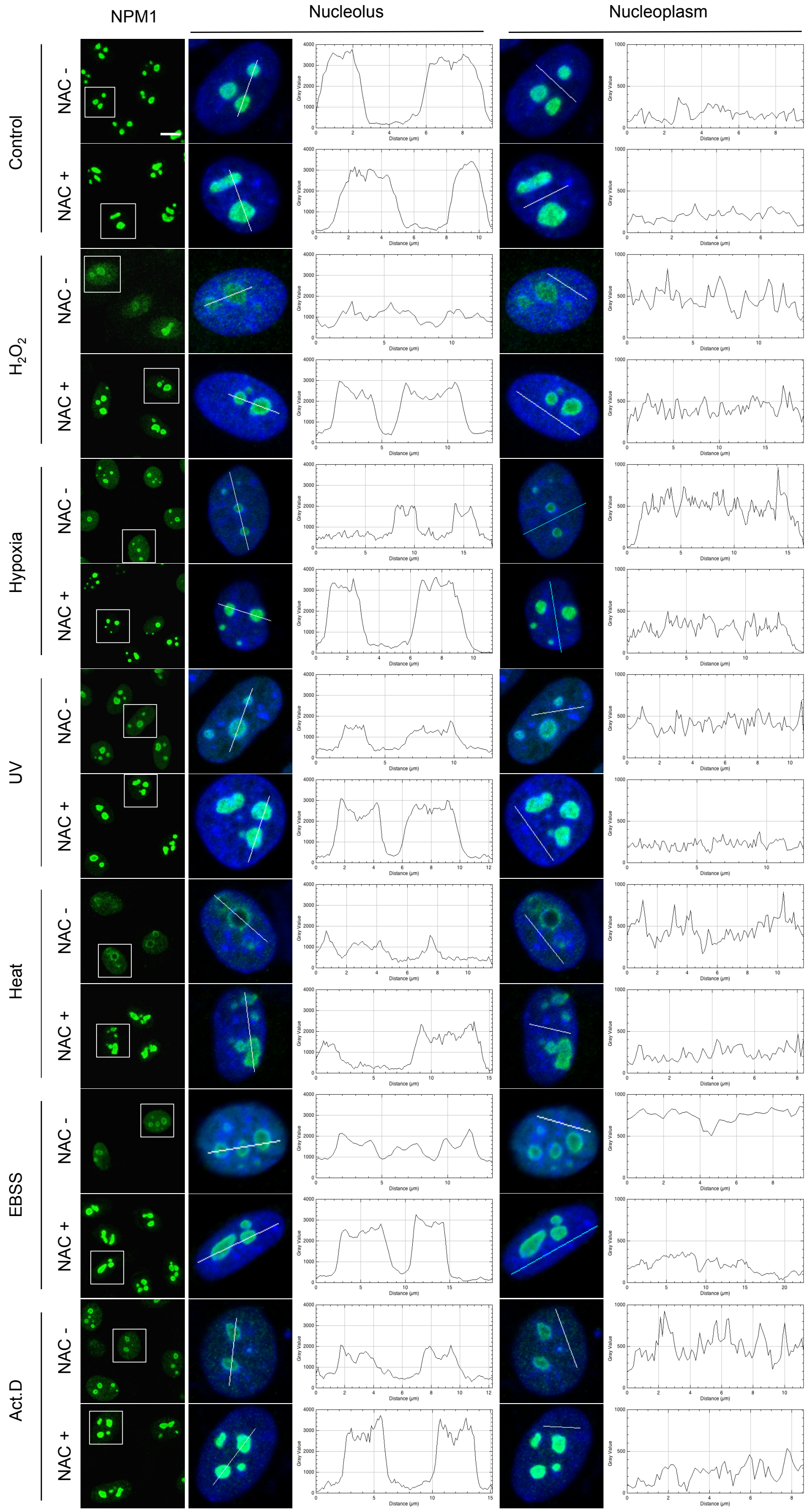
NPM1
Nucleolus
Nucleoplasm
Control
H_2O_2
Hypoxia
UV
Heat
EBSS
Act.D
NAC -
NAC +

b

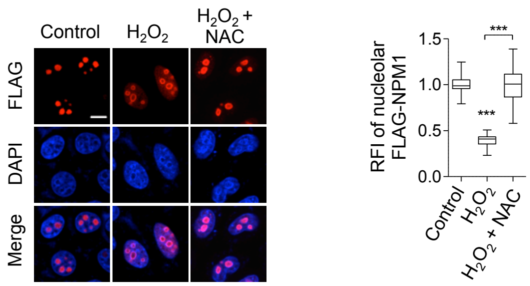

c

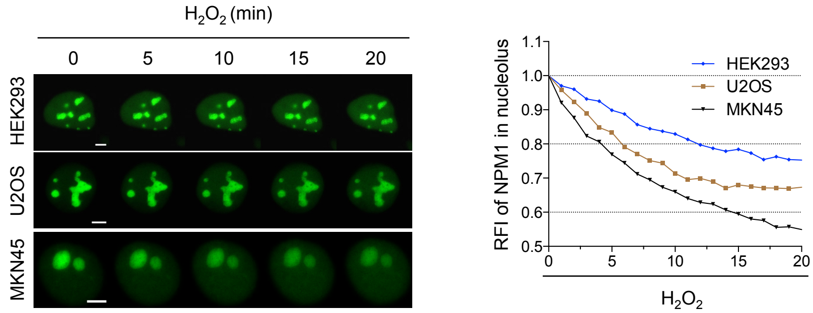

Supplementary Figure 2 | (a) Nucleolar and nucleoplasmic line profiles of NPM1 in representative cells of Fig. 2**a**. **(b)** Translocation of FLAG-NPM1 upon H_2O_2 (500 μM) exposure ± NAC (5 mM) pretreatment in HeLa cells, examined by immunofluorescence using anti-FLAG antibody. RFI of the nucleolar FLAG-NPM1 were displayed. *n*=34 cells each group from three independent experiments. Unpaired *t* test. $P < 0.001$ (***) with respected to treated *vs.* untreated or - NAC *vs.* + NAC cells. **(c)** EGFP-NPM1 nucleolar translocations of 20 min after H_2O_2 (500 μM) treatment in HEK293, U2OS and MKN45 cell lines. Representative images from two independent experiments and RFI of nucleolar NPM1 per minute were displayed. Bars: **c**, 5 μm; **a**, **b**, 10 μm. Data are represented as mean ± s.e.m. This is related to Fig. 2.

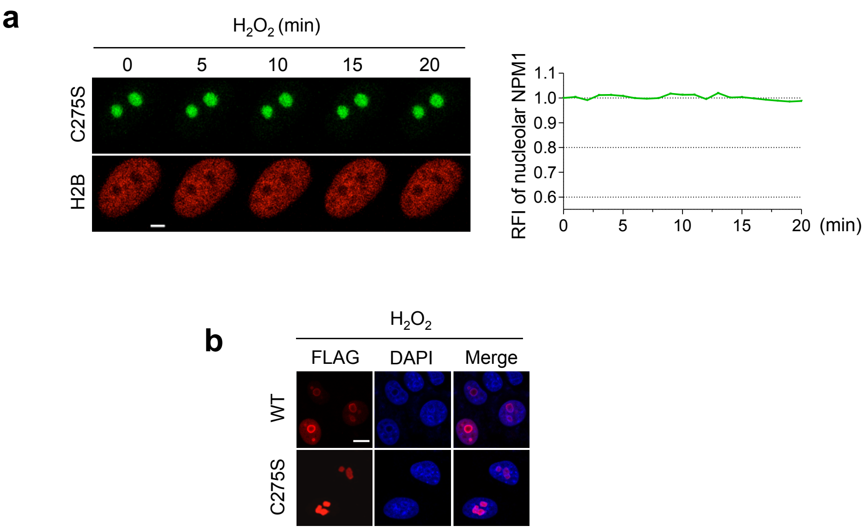

Supplementary Figure 3 | (a) Localization of EGFP-NPM1 mutant C275S and mCherry-H2B in identical cells upon H_2O_2 (500 μM) exposure, visualized by live-cell imaging for 20 min. Representative images and RFI of nucleolar NPM1 per minute were showed. **(b)** Localization of FLAG-NPM1 WT and mutant C275S upon H_2O_2 (500 μM) exposure was examined by immunofluorescence using anti-FLAG antibody. Bars: **a**, 5 μm; **b**, 10 μm. This is related to Fig. 3.

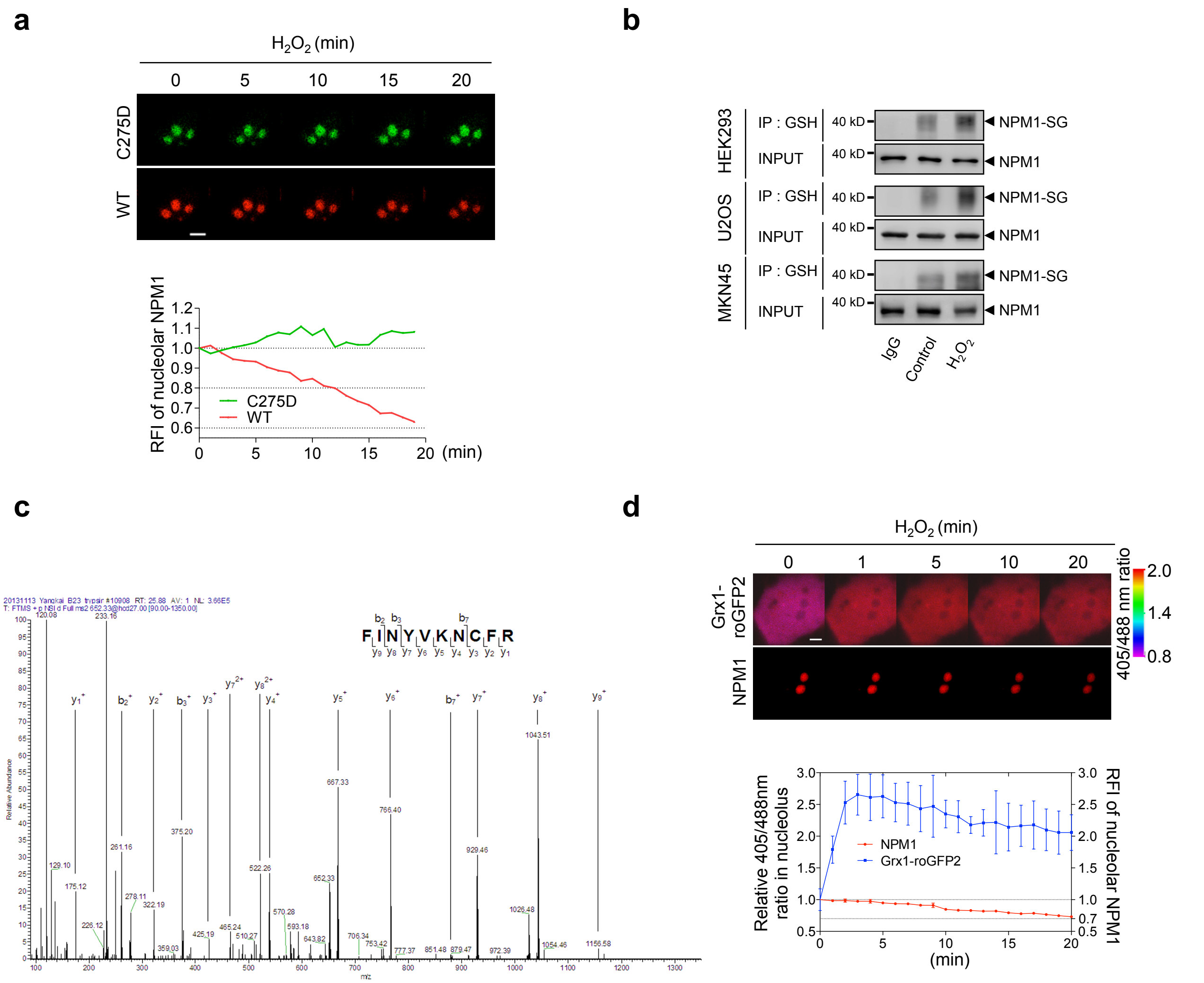

Supplementary Figure 4 | (a) NPM1 mutant C275D bearing negative charge does not localize in the nucleoplasm. Translocation of mCherry-NPM1 WT and EGFP-NPM1 mutant C275D in identical cells upon H_2O_2 (500 μM) exposure, visualized by live-cell imaging for 20 min. Representative images and RFI of nucleolar NPM1 per minute were displayed. **(b)** Endogenous NPM1 S-glutathionylation changes after H_2O_2 (500 μM) treatment in HEK293, U2OS and MKN45 cell lines. Non-reduced co-immunoprecipitation, blotted with anti-NPM1 antibody. **(c)** MS/MS analysis of NPM1 protein derived from non-reduced co-immunoprecipitation. The MS/MS spectrum of the *m/z* y_5^+ (667.3344) was corresponding to the non-modified NPM1 peptide KNCFR, comparing to the S-glutathionylated peptide spectrum 972.4026 in Fig. 4**b**. **(d)** mCherry-NPM1 translocation and GSSG/GSH ratio state (Grx1-roGFP2 biosensor) upon H_2O_2 (500 μM) exposure in the same cells. Concurrent changes of relative 405/488 nm ratio (left *y* axis and blue line) and RFI of nucleolar EGFP-NPM1 (right *y* axis and red line) per minute were plotted. Bars: 5 μm. Data are represented as mean ± s.e.m. This is related to Fig. 4.

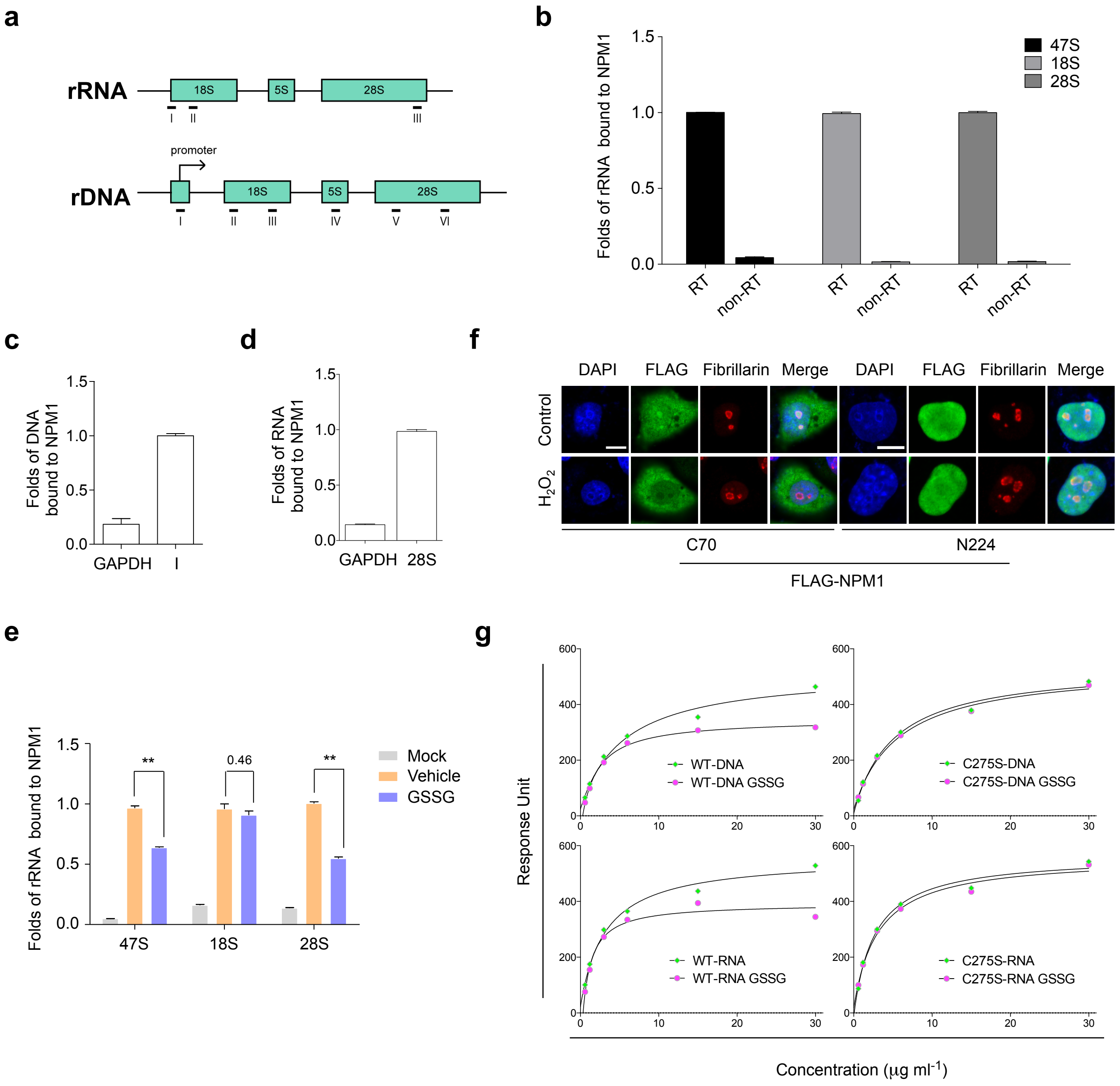

Supplementary Figure 5 | (a) The primers targeted to various regions of rRNA and rDNA. **(b)** In RIP assay, non-reverse-transcribed (non-RT) RNA of Control group was subjected to qPCR experiment, refers to the negative control. **(c)** In ChIP assay, GAPDH which are not expected to be bound with immunoprecipitated FLAG-NPM1, was subjected to qPCR assay. **(d)** Similar to ChIP assay, GAPDH primer was also included in qPCR assay. **(e)** After GSSG (10 mM) was added to the lysis buffer in RIP assays, the quantities of rRNA bound to FLAG-NPM1 WT were assessed. Unpaired *t* test, $P < 0.01$ (**) or showed above the bars. Mock referred to the transfection negative control, whereas Vehicle referred to the treatment negative control. **(f)** The subcellular localization of FLAG-NPM1 truncates C70 and N224. FLAG tagged NPM1 truncates were transfected to HeLa cells before H_2O_2 (500 μM) challenge, examined by immunofluorescence. Fibrillarin served the marker for nucleolar compartment. **(g)** Response units were plotted against protein concentrations. The dissociation constant (K_D) values were calculated with a one-site steady state binding model (see Table 1). Bar. 5 μm. Data are represented as mean ± s.e.m. This is related to Fig. 5 and Fig. 6.

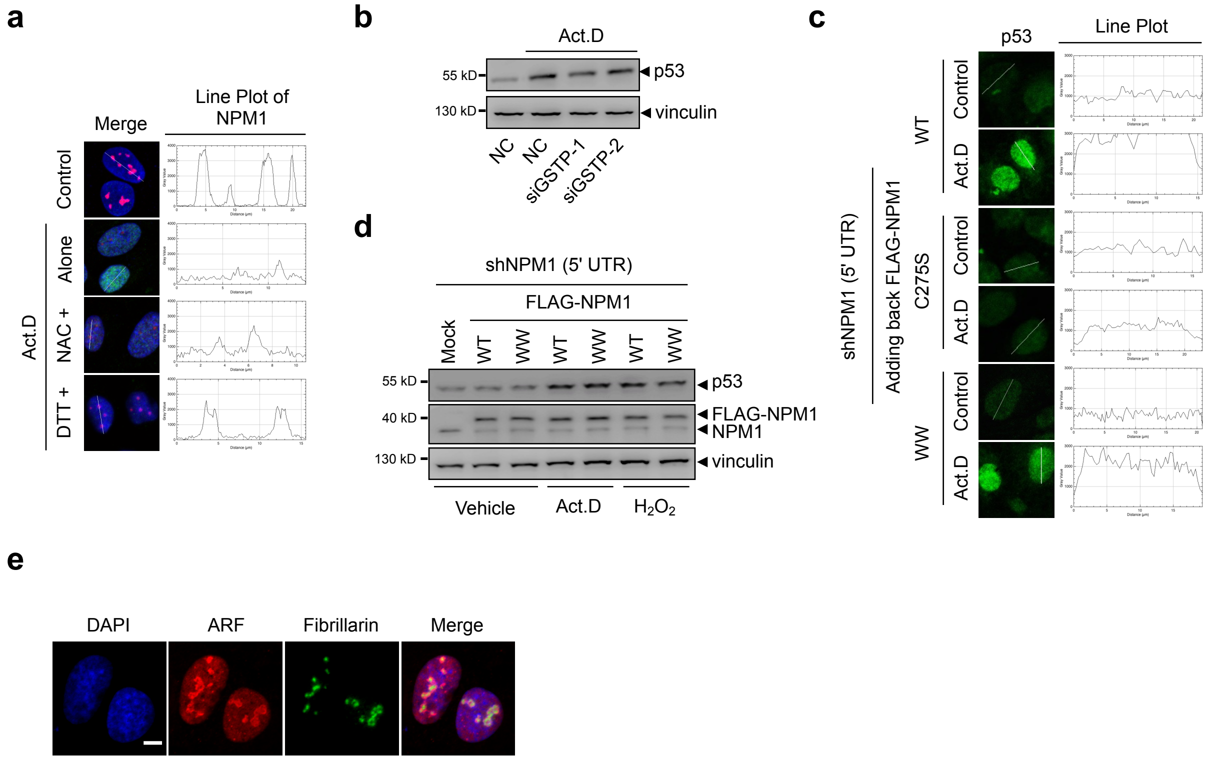

Supplementary Figure 6 | (a) Line profiles of nucleolar NPM1 in representative cells of Fig. 6**a**. **(b)** p53 accumulation after Act.D (8 nM) treatment was attenuated in GSTP silenced cells with siGSTP-1 siRNA oligo (see Fig. 4**c**). **(c)** Line profiles of p53 in representative cells of Fig. 6**c**. **(d)** p53 accumulation after Act.D or H_2O_2 treatment in NPM1-silenced cells which were added back WT or WW mutant of NPM1. Endogenous and exogenous FLAG-NPM1 were bloted with anti-NPM1 antibody. **(e)** The subcellular localization of exogenous HA-ARF were examined by immunofluorescence with anti-HA antibody. Fibrillarin served the marker for nucleolar compartment. Bar. 5 µm. All of experiments were performed in U2OS cells. WW, W288/290A. Data are represented as mean ± s.e.m. This is related to Fig. 7 and Fig. 8.

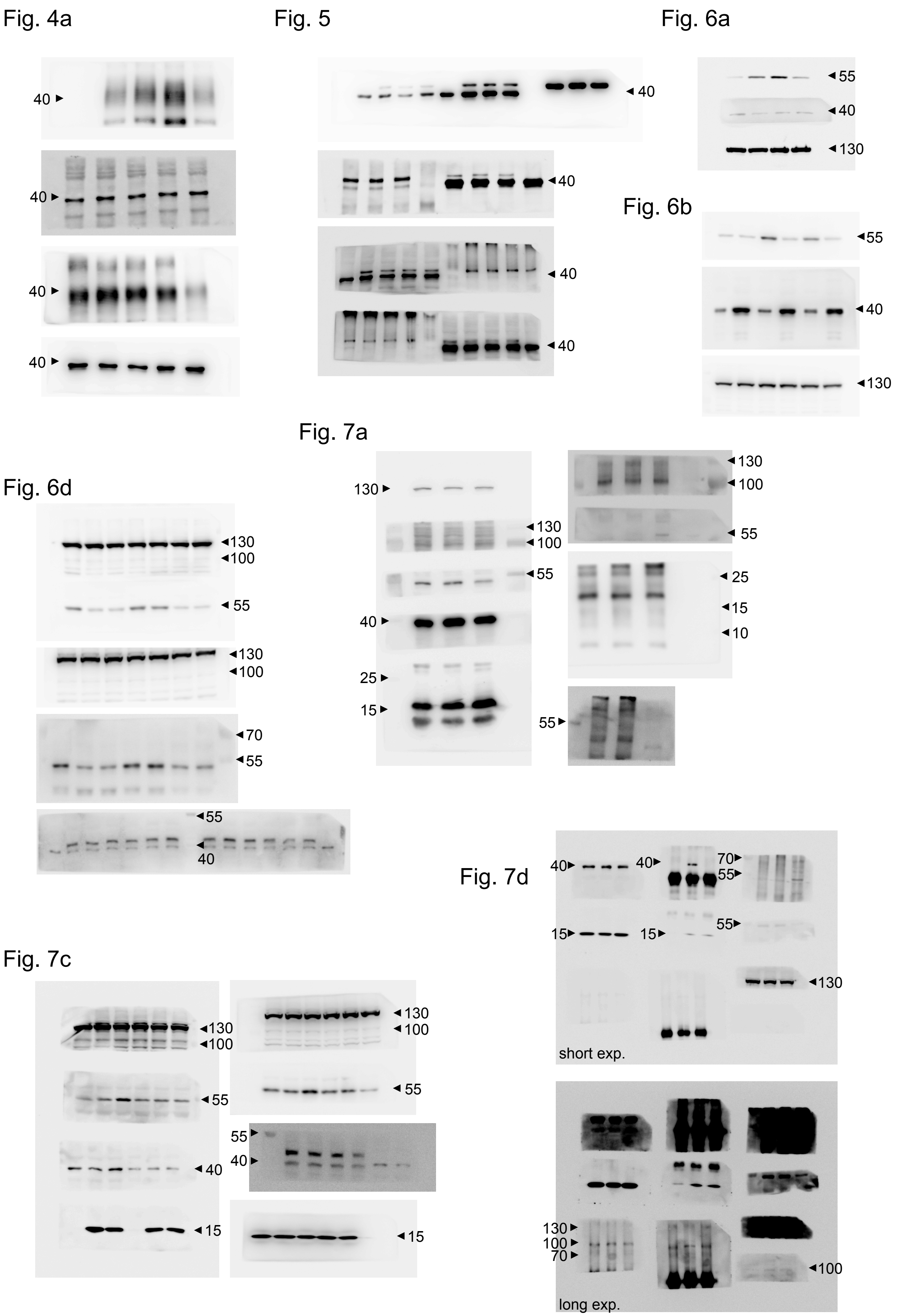

Supplementary Figure 7 | Uncropped versions of all western blots

Supplementary table 1 | Primers used in this study.

Assay	Names	Sequences (5'- 3')
RIP	I-for	cgccgcgctctaccttaccta
	I-rev	taggagaggagcgagcgacca
	II-for	ctgccctatcaactttcgatggtag
	II-rev	ccgtttctcaggctccctctc
	III-for	tgtcggctcttcctatcattgt
	III-rev	acccagctcacgttccctatta
ChIP	I-for	tttcgctccgagtcggc
	I-rev	agcgtgtcagcatataacccg
	II-for	aacggctaccacatccaagg
	II-rev	gggagtgggtaatttgcgc
	III-for	agtgcgggtcataagcttgc
	III-rev	ggtgtgtacaaagggcaggg
	IV-for	ctcttagcggtggatcactcg
	IV-rev	gctagctgcgttcttcatcga
	V-for	gaaactctggtggaggtccg
	V-rev	cggacgaccgatttgcac
	VI-for	agcgttggattgttcaccca
	VI-rev	cggtctaaacccagctcacg
Mutagenesis	C21S	tgtcggcctttagttcactaccgaaaagatagttctg
	C104S	actggccctgaaccactcttcaaccttaagacc
	C275S	gccaaattcatcaattatgtgaagaatagcttccggatgact
	C275D	ggaagccaaattcatcaattatgtgaagaatgacttccggatgactgacc
	Grx1(ss)	gccctcctgctgtacgggctggtgggcttg
	W288A W290A	ggatccttaaagagacttcctcgcctgcgcgagatcttgaatagcctcttgg
Real-time PCR	GSTP-for	gtccaataccatcctgcgtc
	GSTP-rev	gttggtgtagatgagggagatg
	p21-for	tgtcactgtcttgtacccttg
	p21-rev	ggcgtttggagtggtagaa
	PUMA-for	cgacctcaacgcacagtac
	PUMA-rev	cctaattgggctccatctcg
	GAPDH-for	acatcgctcagacaccatg
	GAPDH-rev	tgtagttgaggtcaatgaaggg
